# A New Taxonomic Placement of *Cochylis discerta* (Lepidoptera: Tortricidae) to *Falseuncaria* Supported by Congruent Mitogenomic and Morphological Evidence

**DOI:** 10.1002/ece3.72514

**Published:** 2026-01-06

**Authors:** Yinghui Sun, Huifeng Zhao, John W. Brown, Qiangcheng Zeng, Houhun Li

**Affiliations:** ^1^ College of Life Sciences Dezhou University Dezhou Shandong China; ^2^ Hebei Key Laboratory of Animal Diversity, Langfang Key Laboratory of Bioinformatics, College of Life Science Langfang Normal University Langfang China; ^3^ National Museum of Natural History Washington DC USA; ^4^ College of Life and Geographic Sciences, Key Laboratory of Biological Resources and Ecology of Pamirs Plateau in Xinjiang Kashi University Kashi China; ^5^ College of Life Sciences Nankai University Tianjin China

**Keywords:** Lepidoptera, mitogenome, new combination, phylogeny

## Abstract

In the realm of taxonomic research focused on the Lepidoptera order, our investigation provides evidence supporting the potential reclassification of *Cochylis discerta* Razowski. The complete mitochondrial genomes of four species of Cochylini were newly sequenced: *Aethes alatavica* (Danilevskij), *Cochylis discerta* Razowski, *Cochylis faustana* (Kennel), and *Falseuncaria kaszabi* Razowski. Incorporating these new data, we conducted phylogenomic analyses on the family Tortricidae using Bayesian inference (BI) and maximum likelihood (ML) methods. The results of these analyses are congruent with morphological evaluations, including details of wing patterns, genitalia architecture, and other traits. Taken together, the molecular and morphological evidence supports the provisional transfer of *Cochylis discerta* Razowski to *Falseuncaria* Obraztsov & Swatschek, resulting in *F. discerta* (Razowski), new combination.

## Graphical abstract

By integrating comprehensive mitogenomic sequencing with detailed morphological examinations, we demonstrate a strong case for transferring *Cochylis discerta* from its current genus to *Falseuncaria*. Four complete mitogenomes of Cochylini species are sequenced and analyzed for the first time here.

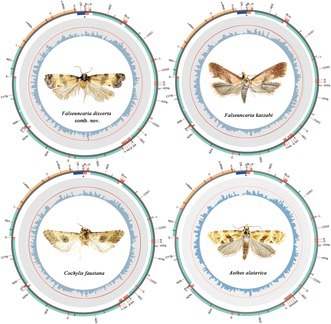

## 1 Introduction

Tortricidae is the largest family of the so‐called microlepidoptera, comprising three subfamilies: Tortricinae, Olethreutinae, and Chlidanotinae. The family is worldwide in distribution, and includes numerous important pests of crops, forests, and ornamental plants, as well as species used for biological control of invasive weeds (Randall et al. 2023) and as experimental models (Roe et al. 2009). The common name “leaf‐roller moths” refers to the fact that the larvae of many species curl or roll leaves of the host plant to create a feeding shelter.

Despite the crucial importance of understanding the phylogeny of Tortricidae for organizing, communicating, and predicting biological features about these economically important insects, our current comprehension of tortricid phylogeny remains somewhat preliminary. Regier et al. sequenced up to 19 genes in 52 tortricid species, and their phylogenetic analyses yielded robust support for the monophyly of the family and several major lineages within it, including the subfamilies Tortricinae and Olethreutinae; Chlidanotinae, however, were shown to be paraphyletic (Regier et al. 2012). Similar results were reported by Fagua et al. (2017, 2018). The inclusion of Cochylini in Tortricidae, which had long been considered a distinct family, was confirmed by Regier et al. (2012). Their analysis suggested that Cochylina (as a subtribe) is a monophyletic group embedded within a broader paraphyletic Euliina (subtribe). However, due to the limited sample size, these results were not particularly robust. There was no comprehensive phylogeny or classification of the genera of Cochylina worldwide until Brown et al. confirmed the hypothesis that Cochylina is indeed a monophyletic group embedded within Euliina (Brown et al. 2019). Their analysis was based on a multi‐loci (mitochondrial and nuclear genes) analysis of 70 species representing 24 genera of Cochylina and eight genera of Euliina, and confirmed the earlier work of Regier et al. (2012).

Cochylini is now widely recognized as one of several tribes that comprise Tortricinae. The taxon was erected by Guenée in 1845 for the type genus *Cochylis* Treitschke, 1829, and currently includes more than 240 genera and 2200 species worldwide, with the greatest diversity in the Palearctic and Neotropical regions (Gilligan et al. 2025). Several species of Cochylini are significant pests of crops, fruit, and forest trees, and are economically significant in agroforestry (Sun and Li 2013a).

Brown et al. recognized six major monophyletic lineages within Cochylina: a *Phtheochroa* Group, a *Henricus* Group, an *Aethes* Group, a *Saphenista* Group, a *Phalonidia* Group, and a *Cochylis* Group. Each group is supported by morphological characters and is consistent with the molecular data (Brown et al. 2019).

Because previous phylogenetic work focused mostly on relationships among and within tribes and subtribes, there has been limited focus on species‐level relationships, and some of the genera are somewhat ill‐defined. The type species of both *Cochylis* and *Falseuncaria* were originally described in *Tortrix*, and the two genera are similar in morphological characteristics.

Razowski described *Cochylis discerta* from males only in 1970 (Razowski 1970), and Sun and Li first reported females of the species in China in 2013 (Sun and Li 2013b). The species can be distinguished from its congeners by its broad transtilla and the absence of a median process in the male genitalia, and by the ring‐shaped sterigma and heavily sclerotized antrum in the female genitalia. However, these morphological features are more similar to those of species of the genus *Falseuncaria* than to those of other species of *Cochylis*. This led to our speculation that *C. discerta* should be transferred to *Falseuncaria* Obraztsov & Swatschek. Hence, we investigated this hypothesis based on molecular data.

Studies based on mitochondrial genomes (mitogenome) have proved more robust than those based on a single locus or several loci (Wang et al. 2019; Deng et al. 2022), so we employed this method to evaluate the generic assignment of *discerta*.

The typical mitogenome of arthropods is a circular, double‐stranded molecule which encodes 37 genes, including 13 protein‐coding genes (PCGs), two ribosomal RNA genes (rRNAs), 22 transfer RNA genes (tRNAs), and an A + T‐rich region (Boore 1999; Cameron 2014). Due to cellular abundance, an absence of introns, rapid evolutionary rate, and a lack of extensive recombination, mitogenome sequences can be easily amplified and have been proven to be a useful source that has been extensively employed in systematics, population genetics and evolutionary biology in the past decades (Curole and Kocher 1999; Simon et al. 2006; Wang 2010; Timmermans et al. 2014; Li et al. 2017). The first tortricid mitogenome was reported in 2006 (*Adoxophyes honmai*); there are currently 40 mitogenomes for Tortricidae (Lee et al. 2006; Son 2011; Zhao et al. 2011; Zhu et al. 2012; Shi et al. 2013; Wu et al. 2013, 2016; Niu et al. 2016; Piper et al. 2016; Zhao et al. 2016, 2021; Fagua et al. 2018; Ding et al. 2020; Xiang 2020; Qi, Sun, et al. 2021; Qi, Zhao, et al. 2021; Yang et al. 2021; Song et al. 2022).

To begin to rectify some of the above issues, we sequenced four complete mitogenomes of Cochylini species, including *Aethes alatavica* (Danilevskij), *C. faustana* (Kennel), *F. discerta* (Razowski) comb. nov. and *F. kaszabi* Razowski. In addition, we conducted a comparative analysis to quantify the genomic organization, nucleotide composition, codon usage, and tRNAs' secondary structure. Moreover, we used the newly sequenced and publicly available mitogenomes to reconstruct a phylogenetic tree to reaffirm the phylogenetic position of Cochylini within Tortricinae.

## 2 Materials and Methods

### 2.1 Specimen Collection

Four Cochylini species collected by light traps in China (Table S1) were preserved in 99.5% ethanol during fieldwork. Samples for DNA extraction were then stored at −20°C in Dezhou University, Shandong, China. Detailed depository information for each specimen is indicated in the systematic section.

### 2.2 Morphological Analyses

The specimens were identified based on the morphological characters. Genitalia were prepared and mounted according to the methods presented by Li (2002). Images of the adults were taken with a Nikon D300 digital camera plus macro lens, and illustrations of the genitalia were prepared by using an Olympus C‐7070 digital camera attached to an Olympus BX51 microscope. Terminology for morphological structures follows Razowski (1987).

### 2.3 DNA Extraction and High Throughput Sequencing

Total genomic DNA was extracted from the muscle tissues of the legs of the adult using the DNeasy Blood and Tissue kit (QIAGEN Sciences, Valencia, CA, USA). The genomic DNA was subsequently pooled with that of other insect species and sequenced using the Illumina Nova6000 (PE150, Illumina, San Diego, CA, USA) platform at Novogene Co. Ltd. (Beijing, China). The 4–6 GB raw data of each species was processed following Zhao et al. (2022) for cleaning the data.

### 2.4 Data Assembly, Annotation, and Analysis

The clean data was assembled by MitoZ v2.4 (Meng et al. 2019) with the default parameters with “MitoZ.py annotate ‐‐genetic_code 5 ‐‐clade Arthropoda”. The python script “circle_check.py” was used to ensure the circular or linear mitogenome. Specifically, to identify the putative overlap region, the script first scans the 3′‐terminal segment of the contig for a unique k‐mer that exactly matches the 5′ terminus. Once this unique anchor is located, the algorithm extends the comparison window base by base toward the 3′ end, tolerating a user‐defined number of mismatches between the 5′ and 3′ counterparts. Extension continues until the mismatch threshold is exceeded or the sequence terminus is reached. At this point, the overlapping stretch is excised, and the trimmed sequence is annotated as “topology = circular”; otherwise, the contig is reported as linear.

All calculations were performed in the high‐performance computing platform at Langfang Normal University. The gene annotation was checked based on the published tortricid mitogenomes to ensure the start and stop positions of genes. The mitogenomic figure was visualized by “MitoZ.py visualize” adding the GC content and base pair sequence depth. The complete mitogenomes of *A. alatavica* , *C. faustana*, *F. discerta* comb. nov. and *F. kaszabi* have been submitted to GenBank (accession numbers: PP407280, PP407286, PP407281, and PP905388, respectively). Nucleotide composition and relative synonymous codon usage (RSCU) of the Cochylini mitogenomes were calculated in MEGA 5 (Tamura et al. 2011). Nucleotide compositional skew was calculated according to the formulas: AT skew = [A − T]/[A + T], GC skew = [G − C]/[G + C] (Perna and Kocher 1995). Across PCGs, the nucleotide diversity *π* of available tortricid PCGs was calculated and visualized in R by using the packages APE v5.8‐1 (https://github.com/emmanuelparadis/ape), pegas v1.3 (https://emmanuelparadis.github.io/pegas.html), and ggplot2 v4.0.0 (https://github.com/tidyverse/ggplot2).

### 2.5 Phylogenetic Analyses

To investigate the phylogenetic implications of the mitogenomes of the new Cochylini sequence data, we reconstructed relationships within Tortricinae using a dataset of the 13 PCGs employing two inference methods, i.e., Bayesian inference (BI) and maximum likelihood (ML). The mitogenomic phylogeny included 21 ingroups (17 publicly available mitogenomes and four newly produced in this study) and 13 Olethreutinae as outgroups (Table 1).

**TABLE 1:** Mitogenomes of Tortricidae and outgroups used in this study.

Subfamily	Tribe	Taxa	Accession no.	References
Olethreutinae	Grapholitini	*Grapholita molesta*	HQ116416	Gong et al. (2012)
Olethreutinae	Grapholitini	*Grapholita dimorpha*	KJ671625	Niu et al. (2016)
Olethreutinae	Grapholitini	*Matsumuraeses phaseoli*	OP575915	Huang et al. (2023)
Olethreutinae	Grapholitini	*Leguminivora glycinivorella*	MH013480	Unpublished
Olethreutinae	Enarmoniini	*Loboschiza koenigiana*	MH013482	Yang et al. (2021)
Olethreutinae	Eucosmini	*Spilonota lechriaspis*	HM204705	Zhao et al. (2011)
Olethreutinae	Eucosmini	*Retinia pseudotsugaicola*	KF498969	Unpublished
Olethreutinae	Olethreutini	*Celypha flavipalpana*	MT548574	Xiang (2020)
Olethreutinae	Olethreutini	*Meiligma* sp.	OP575914	Huang et al. (2023)
Olethreutinae	Olethreutini	*Phiaris dolosana*	MK962620	Li et al. (2019)
Olethreutinae	Olethreutini	*Eudemis lucina*	MK820027	Zhuang et al. (2019)
Olethreutinae	Olethreutini	*Lobesia botrana*	KP677508	Piper et al. (2016)
Olethreutinae	Olethreutini	*Lobesia* sp.	KX621053	Unpublished
Tortricinae	Archipini	*Choristoneura fumiferana*	MG948542	Fagua et al. (2018)
Tortricinae	Archipini	*Choristoneura occidentalis*	MG948541	Fagua et al. (2018)
Tortricinae	Archipini	*Choristoneura biennis*	MG948540	Fagua et al. (2018)
Tortricinae	Archipini	*Choristoneura pinus pinus*	MG944242	Fagua et al. (2018)
Tortricinae	Archipini	*Choristoneura occidentalis*	MG948539	Fagua et al. (2018)
Tortricinae	Archipini	*Choristoneura rosaceana*	MG948544	Fagua et al. (2018)
Tortricinae	Archipini	*Choristoneura conflictana*	MG944241	Fagua et al. (2018)
Tortricinae	Archipini	*Choristoneura murinana*	MG948543	Fagua et al. (2018)
Tortricinae	Archipini	*Choristoneura longicellana*	HQ452340	Fagua et al. (2018)
Tortricinae	Archipini	*Epiphyas postvittana*	KJ508051	Timmermans et al. (2014)
Tortricinae	Archipini	*Adoxophyes honmai*	DQ073916	Lee et al. (2006)
Tortricinae	Archipini	*Adoxophyes orana*	JX872403	Wu et al. (2013)
Tortricinae	Tortricini	*Acleris fimbriana*	HQ662522	Zhao et al. (2016)
Tortricinae	Ceracini	*Cerace xanthocosma*	MT499230	Ding et al. (2020)
Tortricinae	Cochylini	*Aethes alatavica*	PP407280	This study
Tortricinae	Cochylini	*Falseuncaria discerta* comb. nov.	PP905388	This study
Tortricinae	Cochylini	*Falseuncaria kaszabi*	PP407281	This study
Tortricinae	Cochylini	*Cochylis faustana*	PP407286	This study
Tortricinae	Cochylini	*Cochylidia moguntiana*	MW413307	Zhao et al. (2021)
Tortricinae	Cochylini	*Cochylimorpha cultana*	MW413306	Qi, Sun, et al. (2021); Qi, Zhao, et al. (2021)
Tortricinae	Cochylini	*Eugnosta dives*	MW491464	Unpublished

For PCGs, the best DNA model based on Akaike information criterion (AIC) was determined using jModeltest 2.1.7 (Darriba et al. 2012) (Table S7), and those selected models were used by BI with software MrBayes 3.2.6 (Ronquist et al. 2012). To ensure that the average standard deviation of split frequencies was less than 0.01, eight million generations were run with sampling every 1000 generations. Node support was assessed by posterior probabilities (PPs). The ML analyses were performed using RAxML v8 (Stamatakis 2014), selecting the best model and constructing phylogenetic trees automatically using 1000 non‐parametric bootstrap replicates (BS).

Tracer v1.6 (http://tree.bio.ed.ac.uk/software/tracer/) was used to check the likelihoods of all parameters of BI analyses to ensure the effective sample size (ESS) values greater than 200. The consensus tree was calculated by discarding the first 25% of trees. To verify the consistencies of the topologies, both BI and ML analyses were repeated three times, and the phylogenetic trees were visualized by Figtree v.1.4.4 (http://tree.bio.ed.ac.uk/software/figtree/).

## 3 Results

### 3.1 Systematics

**FIGURE 1:**
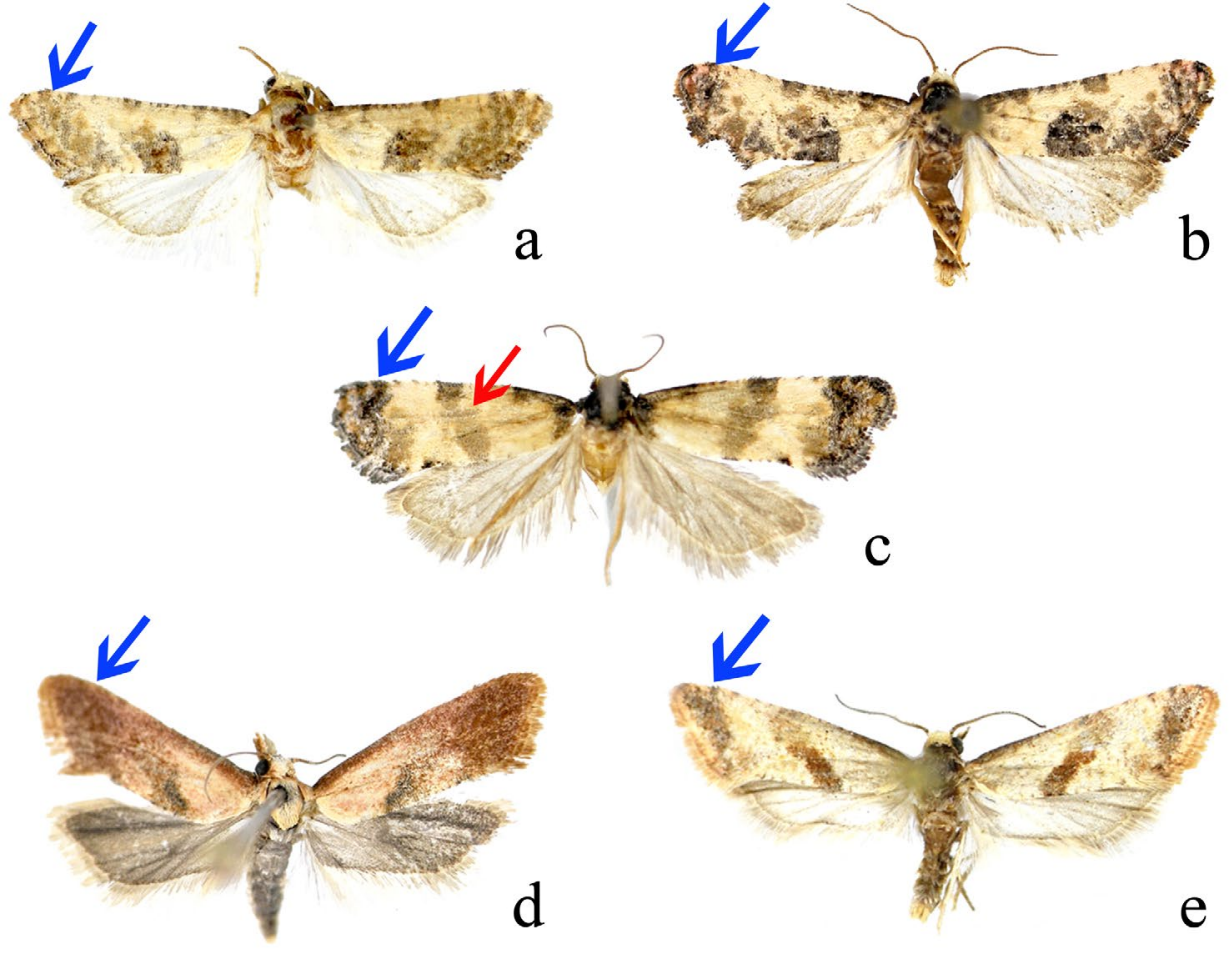
Adults of *Cochylis* and *Falseuncaria* spp. (a) *Cochylis faustana* (Sun and Li 2013b); (b) *Cochylis posterana hyrcana* (Sun and Li 2013b); (c) *Falseuncaria discerta* comb. nov. (Sun and Li 2013b); (d) *Falseuncaria kaszabi* (this study); (e) *Falseuncaria ruficiliana* (Sun and Li 2012). The red arrow indicates the thin median fascia and the blue arrows indicate the subapical fascia.

**FIGURE 2:**
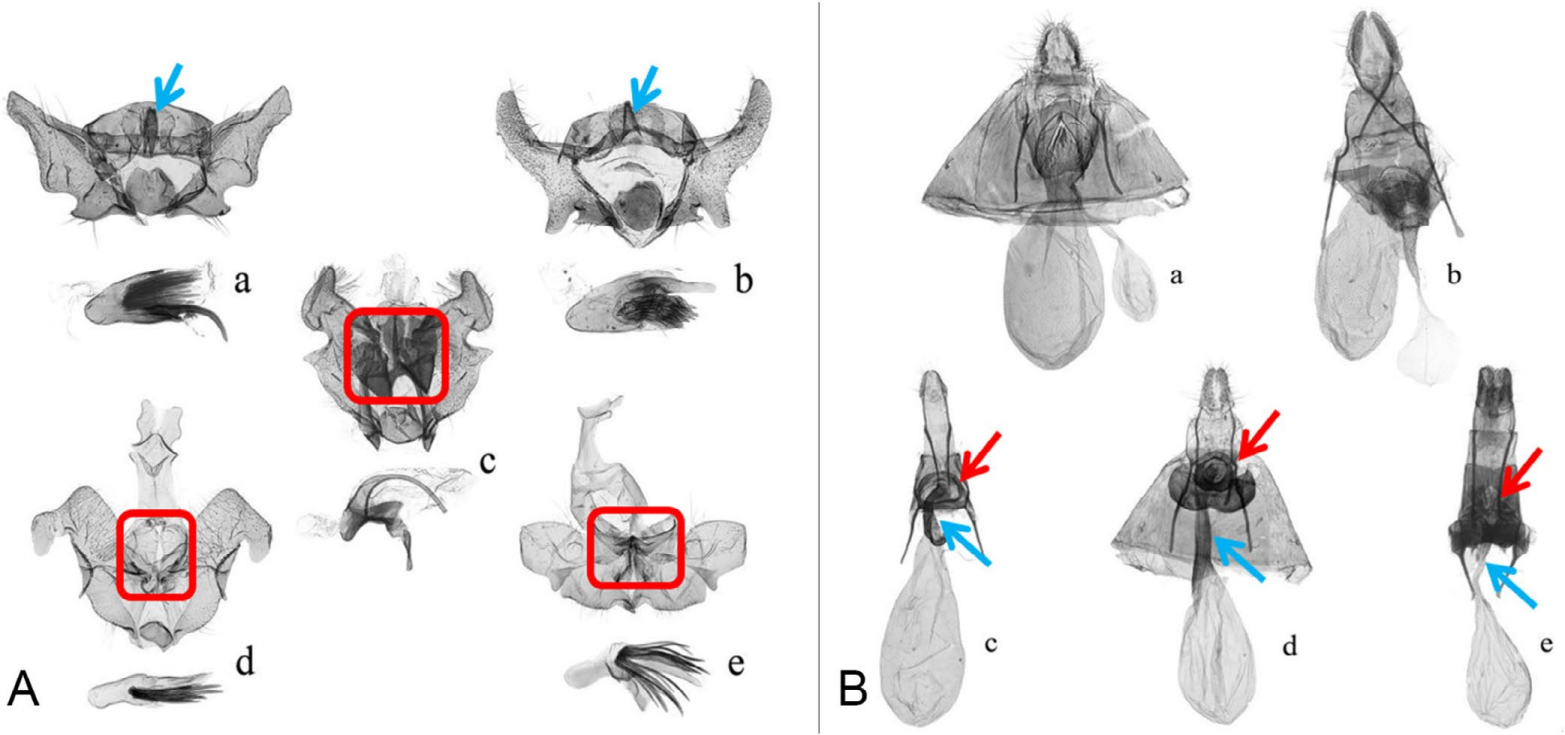
Male genitalia (A) and female genitalia (B) of *Cochylis* and *Falseuncaria* spp. (a) *Cochylis faustana* (Sun and Li 2013b); (b) *Cochylis posterana hyrcana* (Sun and Li 2013b); (c) *Falseuncaria discerta* comb. nov. (Sun and Li 2013b); (d) *Falseuncaria kaszabi* (this study); (e) *Falseuncaria ruficiliana* (Sun and Li 2012). In (A), the red boxes indicate the broad transtilla, and the blue arrows indicate the median process. In (B), the red arrows indicate the ring‐shaped sterigma, and the blue arrows indicate the slender ductus bursae.

To further investigate the relationship among the species of these two genera, several species were selected from each for morphological comparison. Images of the adults and genitalia are provided (Figures 1 and 2).

The forewing pattern provides limited support for the transfer of discerta to *Falseuncaria*. In *Falseuncaria* (Figure 1d,e) the media fascia is typically well defined, parallel‐sided, and characteristically angled from the costa to the hind margin. The median fascia of *discerta* is slightly broader than that of the other species of *Falseuncaria* but narrower than that of *Cochylis* species (Figure 1a,b). In the male genitalia, the transtilla of *discerta*. is broad and the median process is absent (Figure 2Ac). In the female genitalia of *discerta*, the sterigma is ring‐shaped and the ductus bursae is slender (Figure 2Bc). Based on these morphological features, *discerta* appears to be more appropriately placed in *Falseuncaria* than in *Cochylis*. Furthermore, as presently defined *Cochylis* is undoubtedly polyphyletic (Brown et al. 2019). Therefore, the removal of *discerta* from *Cochylis* brings the latter genus one step closer to monophyly.

#### 3.1.1 Checklist and Distributions of the Chinese Species in *Cochylis*

1. *Cochylis atricapitana* (Stephens, 1852)

*Eupoecilia atricapitana* Stephens, 1852: 80. Type locality: England.

Distribution. China (Xinjiang), Europe.

2 *Cochylis defessana* Mann, 1861

*Cochylis defessana* Mann, 1861: 185. Type locality: Turkey.

Distribution. China (Xinjiang), Iran, Turkey.

3 *Cochylis dubitana* (Hübner, [1796–1799])

*Tortrix dubitana* Hübner, [1796–1799]: pl. 12, fig. 71. Type locality: Europe.

Distribution. China (Heilongjiang), Europe.

4 *Cochylis faustana* (Kennel, 1919)

*Phalonia faustana* Kennel, 1919: 73. Type locality: Russia.

Distribution. China (Inner Mongolia, Xinjiang), Russia.

5 *Cochylis hybridella* (Hübner, [1810–1813])

*Tinea hybridella* Hübner, [1810–1813]: pl. 51, fig. 351. Type locality: Germany.

Distribution. Northern China, Korea, Japan, Europe.

6 *Cochylis piana* (Kennel, 1919)

*Phalonia piana* Kennel, 1919: 75. Type locality: Russia.

Distribution. China (Inner Mongolia, Liaoning, Shaanxi, Xinjiang), Afghanistan, Iran, Russia.

7 *Cochylis posterana hyrcana* (Toll, 1948)

*Phalonia posterana hyrcana* Toll, 1948: 112. Type locality: Iran.

Distribution. China (Gansu, Xinjiang), Iran.

8 *Cochylis psychrasema* (Meyrick, 1937)

*Phalonia psychrasema* Meyrick, 1937: 171. Type locality: China.

Distribution. China (Yunnan).

9 *Cochylis roseana* (Haworth, 1811[1812])

*Tortrix roseana* Haworth, 1811[1812]: 401. Type locality: United Kingdom.

Distribution. China (Gansu), Iran, Europe.

10 *Cochylis triangula* Sun *et* Li, 2013

*Cochylis triangula* Sun *et* Li, 2013: 92. Type locality: China.

Distribution. China (Guizhou, Yunnan).

#### 3.1.2 Checklist and Distributions of the Chinese Species in *Falseuncaria*

1. *Falseuncaria brunnescens* Bai, Guo *et* Guo, 1996

*Falseuncaria brunnescens* Bai, Guo *et* Guo, 1996: 191. Type locality: China.

Distribution. China (Shanxi).

2 *Falseuncaria degreyana* (McLachlan, 1869)

*Eupoecilia degreyana* McLachlan, 1869: 91. Type locality: Norfolk Island.

Distribution. China (Xinjiang), Mongolia, Europe.

3 *Falseuncaria discerta* Razowski, 1970 comb. nov.

*Cochylis discerta* Razowski, 1970: 431. Type locality: Mongolia.

Distribution. China (Gansu, Inner Mongolia, Shanxi), Mongolia.

4 *Falseuncaria kaszabi* Razowski, 1966

*Falseuncaria kaszabi* Razowski, 1966: 505. Type locality: Mongolia.

Distribution. China (Gansu, Inner Mongolia, Ningxia, Qinghai, Shaanxi), Mongolia.

5 *Falseuncaria lechriotoma* Razowski, 1970

*Falseuncaria lechriotoma* Razowski, 1970: 437. TL: Mongolia.

Distribution. China (Hebei), Mongolia.

6 *Falseuncaria ruficiliana* (Haworth, 1811[1812])

*Tortrix ruficiliana* Haworth, 1811[1812]: 402. Type locality: United Kingdom.

Distribution. China (Xinjiang), Europe.

### 3.2 Mitogenome Organization and Nucleotide Composition of Cochylini

**FIGURE 3:**
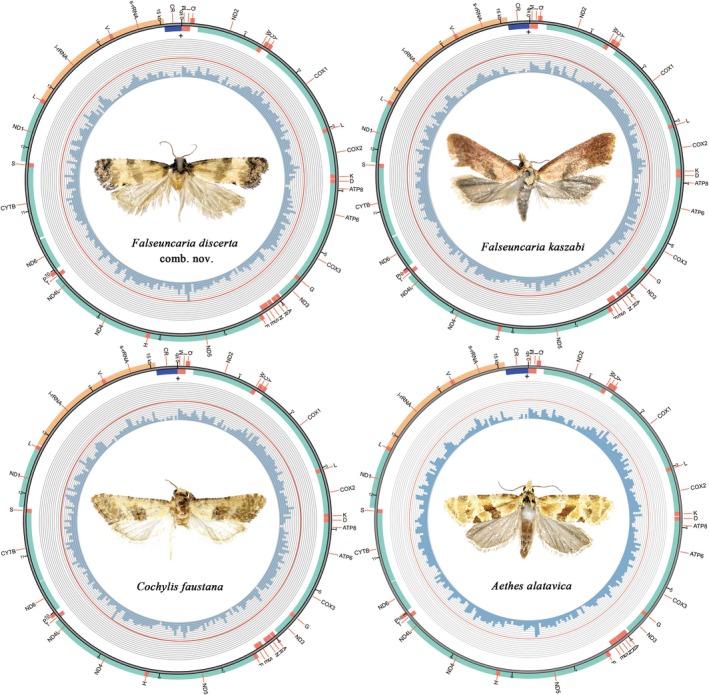
Complete mitochondrial genomes of four species for Cochylini. The inner circle indicates the GC content, the outer circle shows the arrangement of the genes: green for the CDS, red for tRNAs, orange for rRNAs, and purple for the control region.

The complete mitogenomes of the four species of Cochylini investigated here were found to be composed of circular double‐stranded molecules of mildly varying sizes. The annotations for the mitogenomes of the four Cochylini species are shown in Tables S2–S5, and the circular maps of the mitogenomes of the four species are shown in Figure 3, and the coverage of each site of the newly sequenced mitogenomes is shown in Figure S1. Each mitogenome contains the typical set of 37 genes, including 13 PCGs, 22 tRNAs, two rRNAs and an A + T rich region. The majority strand (J‐strand) encoded 23 genes (9 PCGs, 14 tRNAs), while the remaining genes were located on the minority strand (N‐strand) (four PCGs, eight tRNAs, and two rRNAs) (Figure 3, Tables S2–S5). The sizes were as follows: *A. alatavica* 15,582 bp, *C. faustana* 15,427 bp, *F. discerta* comb. nov. 15,330 bp, and *F. kaszabi* 15,426 bp.

In the whole mitogenomes of the four Cochylini species, the nucleotide composition indicated a strong A and T bias, and the A + T% content ranged from 80.2% (in *F. kaszabi*), 80.4% (in *A. alatavica* and *F. discerta* comb. nov.) to 80.8% (in *C. faustana*) (Table S6). Comparing the AT content of the whole mitogenome, PCGs, tRNAs, rRNAs, and control region, the control region was the highest, while the PCGs were the lowest for all the four species of Cochylini (Table S6). The AT skew of the whole mitogenome within the four Cochylini species ranged from 0.007 to 0.013, and GC skew ranged from −0.187 to −0.166.

### 3.3 Protein‐Coding Genes of Cochylini

**FIGURE 4:**
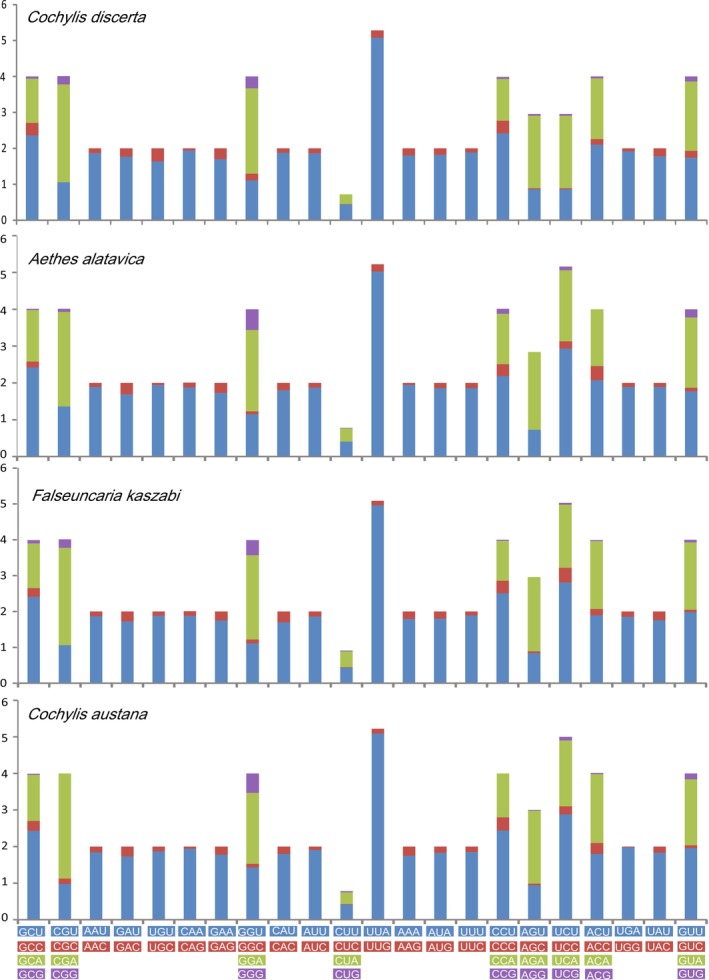
Relative synonymous codon usage (RSCU) in the PCGs of the new sequenced Cochylini mitogenomes. Codon families are indicated below the X axis.

The lengths of PCGs within Cochylini species varied between 156 bp for ATP8 and 1734 bp for ND5, with total PCG lengths spanning from 11,152 to 11,187 bp. Analysis of the four Cochylini mitogenomes revealed consistent start and stop codon usage patterns, as outlined in Tables S2–S5. All PCGs were found to initiate with ATN codons, except for COX1, which uniquely started with CGA. Termination codons were standardized across 8–9 PCGs, with TAA being the most frequent, while COX1, COX2, ATP8, ND4, and ND5 were exceptional in terminating with a single T or TA residue. It is noteworthy that truncated termination codons, frequently observed in metazoan mitogenomes, are post‐transcriptionally modified to complete TAA stop codons through polyadenylation. The RSCU values for the four Cochylini species are presented in Figure 4. The codons UUA‐Leu2, UCU‐Ser2, GGA‐Gly, and UCA‐Ser2 were found to be the most frequently utilized in the Cochylini mitogenome, whereas CGC‐Arg was entirely absent from all the four species; moreover, AGG‐Ser1 was absent from *A. alatavica* and *F. kaszabi*, AGC‐Ser1, ACG‐Thr, and CUC‐Leu1 were absent in *A. alatavica* , CCG‐Pro was absent from *C. faustana*, and CUG‐Leu1 was absent from *F. discerta* comb. nov. Figure S2 shows the results of a sliding window analysis of nucleotide diversity (*π*) across the 13 protein‐coding genes (PCGs) of the mitogenome in Tortricidae. The nucleotide diversity varies significantly among genes, with ND6 exhibiting the highest average π value, indicating the highest level of sequence divergence. In contrast, ND5 shows the lowest π value, suggesting it is the most conserved among the PCGs. Other genes with relatively high nucleotide diversity include ATP8, ND2, ND3, ND4, and COX3, while COX1, COX2, ND1, ND4L, and CYTB show relatively low diversity.

### 3.4 RNA Genes of Cochylini

The 22 tRNAs molecules identified across the four Cochylini species are presented in Figures S3–S6, with total tRNA lengths of 1468, 1465, 1469, and 1484 bp. These tRNAs exhibited size variability ranging from 60 to 73 bp, as detailed in Tables S2–S5. Secondary structural analysis revealed that 19 tRNAs could be folded into the canonical clover‐leaf conformation, with the exception of trnS1 (AGN), which displayed a notable absence of the dihydrouridine (DHU) stem that such structural deviations are well documented in Cochylini and other moth mitogenomes (Qi, Sun, et al. 2021; Yang et al. 2021) (Figures S3–S6), and trnI and trnT loss a TψC loop (Figures S3–S6), which is reported for the first time in Tortricidae. Conservation of secondary structure was observed in the anticodon loop (7 nucleotides), anticodon stem (mostly are 5 bp, except trnS2 which only 3 bp), and acceptor stem (7 bp, except the 5 bp in trnF of *C. faustana*). However, variability was evident in the lengths of the DHU (3–4 bp) and TψC (4–5 bp) stems, except for trnS1 (Figures S3–S6). Additionally, Figures S3–S6 illustrate the presence of mismatched base pairs within tRNA stems (trnS2 of the four species and trnL2 in *F. discerta* comb. nov.), which may be corrected through post‐transcriptional editing mechanisms (Lavrov et al. 2000).

In the four examined Cochylini species, both the large‐subunit ribosomal RNA (l‐rRNA) and the small‐subunit ribosomal RNA (s‐rRNA) genes were localized on the N‐strand (Figure 3 and Tables S1–S4). The l‐rRNA gene exhibited a length variation from 1,388 bp in *F. discerta* comb. nov. to 1,415 bp in *C. faustana*. Similarly, the s‐rRNA gene ranged from 811 bp in *F*. comb. nov. to 838 bp in *A. alatavica* . Nucleotide composition analysis revealed both positive AT skew and GC skew in the large and small rRNAs.

### 3.5 Control Region of Cochylini

The control region, also referred to as the A + T‐rich region, is typically the most extensive non‐coding segment in animal mitogenomes. In Cochylini mitogenomes, this region is positioned between the s‐rRNA and trnM genes. The length of the control region varies: *A. alatavica* exhibits 473 bp, *C. faustana* is 346 bp, *F. kaszabi* 318 bp, and *F. discerta* comb. nov. 274 bp. These values align with the average control region length of approximately 494 bp observed across the published tortricid mitogenomes (172–771 bp) (Son 2011; Qi, Sun, et al. 2021; Qi, Zhao, et al. 2021; Yang et al. 2021; Zhao et al. 2021). Nucleotide composition analysis reveals a remarkably high A + T content in this region, ranging from 94.5% in *C. faustana* to 95.9% in *F. kaszabi*.

### 3.6 Phylogenetic Analysis

In the tree based on the PCGs dataset, the monophyly of the two subfamilies, Tortricinae and Olethreutinae, was well supported, as has been shown in previous morphology‐ and molecular‐based analyses (Regier et al. 2012; Brown et al. 2019; Qi, Sun, et al. 2021; Qi, Zhao, et al. 2021). The four tribes of Tortricinae included in our analyses (i.e., Archipini, Ceracini, Cochylini, and Tortricini) form a monophyletic group.

**FIGURE 5:**
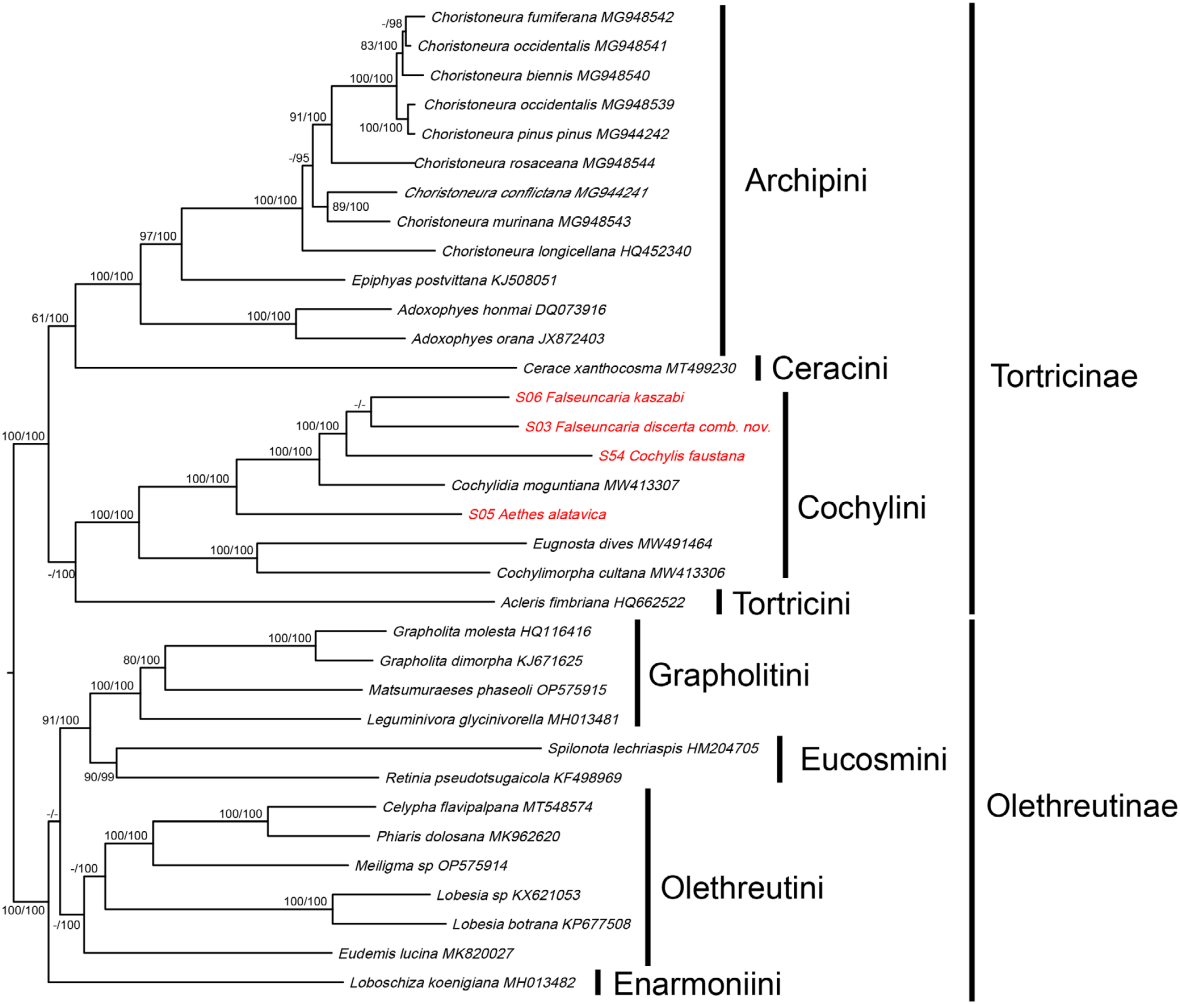
Phylogenetic trees constructed by ML methods based on the dataset of PCGs; both ML and BI analyses show the same topology. The values above the branches are bootstrap support values (BS) and Bayesian posterior probabilities (PPs) BS values lower than 50 and PPs lower than 95 are shown as dashes. Red tips show the taxa sequenced in this study.

Within Cochylini, support for the transfer of *C. discerta* to *Falseuncaria* is provided by the high BS value (BS = 73), although PPs are not as convincing (Figure 5). Additionally, the phylogenetic tree supports the sister relationship between *Falseuncaria* and *Cochylis* with BS = 93 and PP = 100. Although Brown et al. (Regier et al. 2012), identified *Cochylidia* as the sister to *Falseuncaria*, they stated that the relationship was not particularly compelling based on morphology. Based on DNA barcodes alone, *Falseuncaria* was portrayed as embedded within a polyphyletic *Cochylis* (Brown et al. 2019). Hence, its position remains somewhat ambiguous.

## 4 Discussion

Our ML and Bayesian analyses are highly consistent with currently proposed phylogenetic relationships (Figure 5) within Tortricidae (Regier et al. 2012; Fagua et al. 2017). Our results support a major split of the subfamily Tortricinae into two sister lineages, one consisting of Archipini plus Ceracini, and the other comprising Cochylini and Tortricini. Although Tortricini and Cnephasiini have been shown to represent sister taxa, in the absence of mitogenomes for Cnephasiini, our results are at least congruent with previous studies (Regier et al. 2012), and in future the mitogenomes of Cnephasiini should be incorporated to test the relationships within Tortricinae. As for the Olethreutinae, our results show a basal split into two sister lineages, one consisting of Grapholitini plus Eucosmini, and the other comprising Olethreutini. These results likewise are compatible with previous studies of Regier et al. (2012) even though complete mitogenes are unavailable for a couple of Olethreutine tribes.

At the generic level, *Cochylis* and *Falseuncaria* are closely related clades and almost certainly monophyletic. The two genera shared some conspicuoue synapomorphic. *Falseuncaria* is differentiated from *Cochylis* by the broad transtilla and the absence of a median process in the male genitalia, and the ring‐shaped sterigma and heavily sclerotized antrum in the female genitalia (Sun and Li 2013b). The results of the molecular and morphological analyses reinforce one another, provisionally supporting the new combination of *F. discerta* comb. nov.

In the current context of rapid biotechnology, the cost of mitogenome sequencing has been significantly reduced, which provides an unprecedented opportunity to use mitochondrial data for taxonomic research. Compared with traditional morphological classification methods, mitogenome data can effectively make up for the limitations of morphological characteristics in the definition of species and the determination of phylogenetic relationships due to its high resolution, rich genetic information and relatively stable characteristics. As sequencing technology continues to advance and costs further decrease, large‐scale access to mitogenome data will become more convenient and economical. In the future, we are expected to see more studies using this powerful tool to deeply analyze the long‐standing taxonomic problems due to similar morphological characteristics or complex variation, so as to promote the development of biological taxonomic systems in a more accurate and objective direction, and provide a solid scientific foundation for biodiversity conservation and rational use of resources.

## 5 Conclusions

In this study, we reported the mitogenome sequences of *A. alatavica* , *C. faustana*, *F. discerta* comb. nov., and *F. kaszabi*. Compared to previously reported mitogenomes of Tortricidae, the newly sequenced Cochylini complete mitogenomes are conserved in gene organization, base composition, codon usage of PCGs and secondary structures of tRNAs. The phylogenetic analyses inferred from PCGs produced a well‐resolved framework for the relationships of Tortricidae compatible with that of previous phylogenetic studies on the family. Also, the position of *F. discerta* comb. nov. was confirmed. Moreover, it suggested that mitogenomic data are useful for further resolving phylogenetic issues within Cochylini. The results of our analyses suggest that extensive samples of multiple taxa and larger scale analyses with more nuclear markers, or even a whole genome will undoubtedly be helpful toward reconstructing a comprehensive framework of the tortricid phylogeny.

## Author Contributions

**Yinghui Sun:** conceptualization (lead), data curation (supporting), funding acquisition, methodology (lead), writing – original draft (equal). **Huifeng Zhao:** data curation (lead), writing – original draft (equal). **John W. Brown:** writing – review and editing (equal). **Qiangcheng Zeng:** writing – review and editing (equal). **Houhun Li:** conceptualization (supporting), writing – review and editing (equal).

## Conflicts of Interest

The authors declare no conflicts of interest.

## Supporting information


**Figure S1:** Coverage plots of the four mitogenomes newly sequenced by this study.
**Figure S2:** Sliding‐window nucleotide diversity of conserved amino‐acid sites across PCGs of Tortricidae.
**Figure S3:** Secondary structures of 22 transfer RNAs in *Aethes alatavica*.
**Figure S4:** Secondary structures of 22 transfer RNAs in *Cochylis faustana*.
**Figure S5:** Secondary structures of 22 transfer RNAs in *Falseuncaria discerta* comb. nov.
**Figure S6:** Secondary structures of 22 transfer RNAs in *Falseuncaria kaszabi*.
**Table S1:** Collecting information of specimens in present study.
**Table S2:** Mitogenome organization of *Aethes alatavica*.
**Table S3:** Mitogenome organization of *Cochylis faustana*.
**Table S4:** Mitogenome organization of *Falseuncaria discerta* comb. nov.
**Table S5:** Mitogenome organization of *Falseuncaria kaszabi*.
**Table S6:** Nucleotide composition of mitochondrial genomes of four Cochylini species.
**Table S7:** The best substitute DNA model in Tortricidae using jModeltest.

## Data Availability

All sequences were deposited in GenBank under the accession numbers of PP407280, PP407286, PP407281, and PP905388.
