# Supplementary material for: A New Taxonomic Placement of Cochylis discerta (Lepidoptera: Tortricidae) to Falseuncaria Supported by Congruent Mitogenomic and Morphological Evidence
Source: Ecol Evol. 2026 Jan 6;16(1):e72514. doi: 10.1002/ece3.72514 (PMC12771650; doi:10.1002/ece3.72514)
Supplement: Supplementary file 1 — Figure S1: Coverage plots of the four mitogenomes newly sequenced by this study. Figure S2: Sliding‐window nucleotide diversity of conserved amino‐acid sites across PCGs of Tortricidae. Figure S3: Secondary structures of 22 transfer RNAs in Aethes alatavica. Figure S4: Secondary structures of 22 transfer RNAs in Cochylis faustana. Figure S5: Secondary structures of 22 transfer RNAs in Falseuncaria discerta comb. nov. Figure S6: Secondary structures of 22 transfer RNAs in Falseuncaria kaszabi. Table S1: Collecting information of specimens in present study. Table S2: Mitogenome organization of Aethes alatavica. Table S3: Mitogenome organization of Cochylis faustana. Table S4: Mitogenome organization of Falseuncaria discerta comb. nov. Table S5: Mitogenome organization of Falseuncaria kaszabi. Table S6: Nucleotide composition of mitochondrial genomes of four Cochylini species. Table S7: The best substitute DNA model in Tortricidae using jModeltest. [file ECE3-16-e72514-s001.zip › 20251021-revised-supplementary.docx]

Supplementary materials


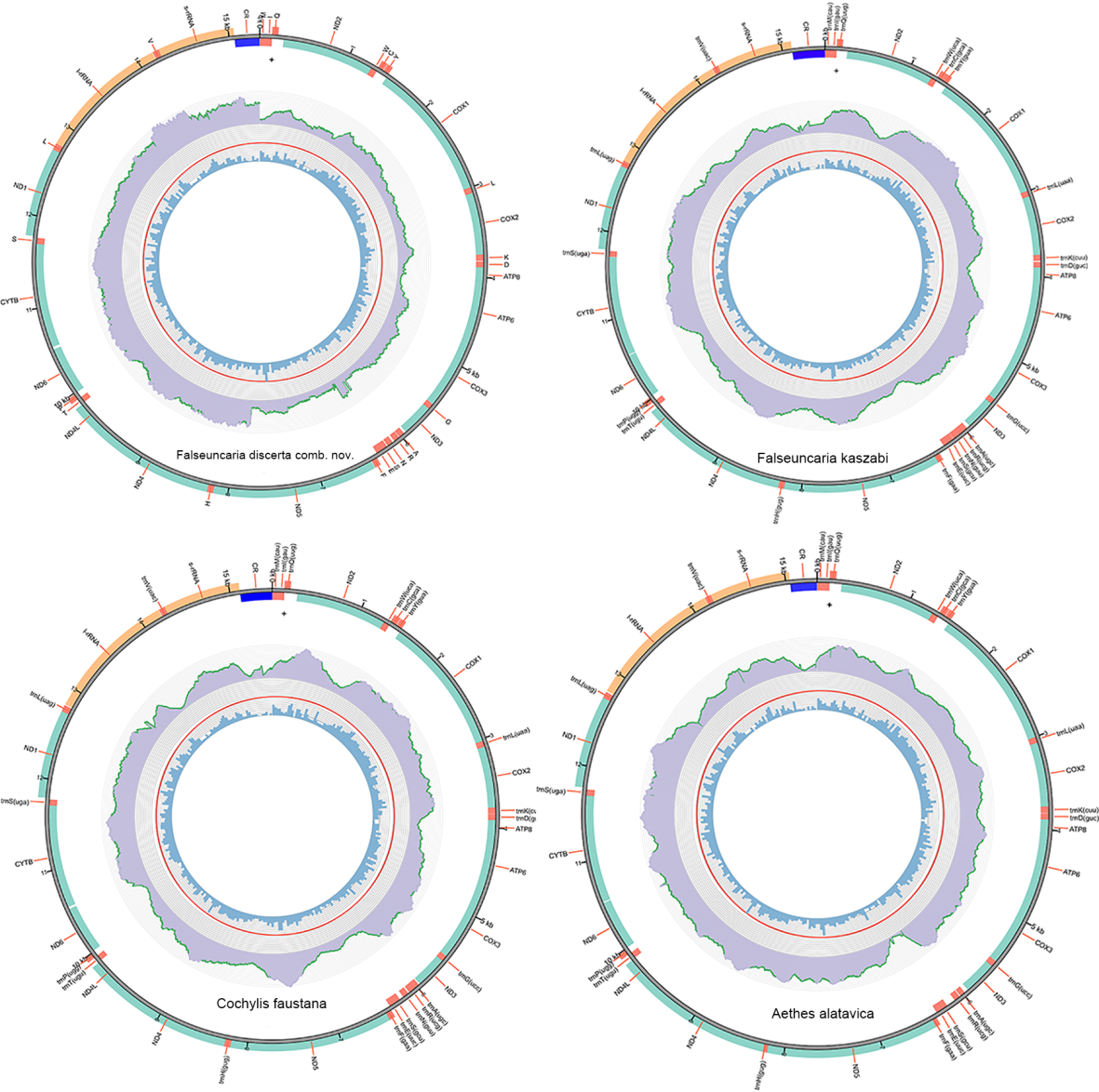


Figure S1. Coverage plots of the four mitogenomes newly sequenced by this study.


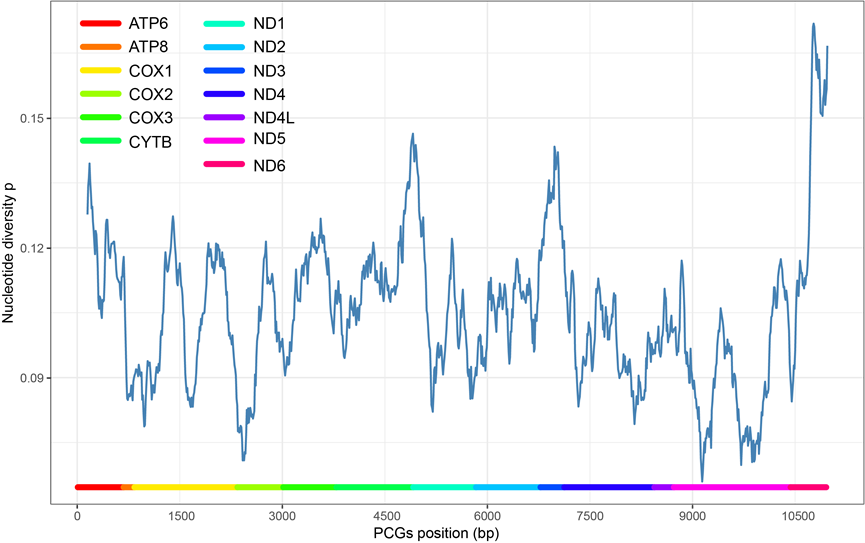


Figure S2. Sliding-window nucleotide diversity of conserved amino-acid sites across PCGs of Tortricidae.


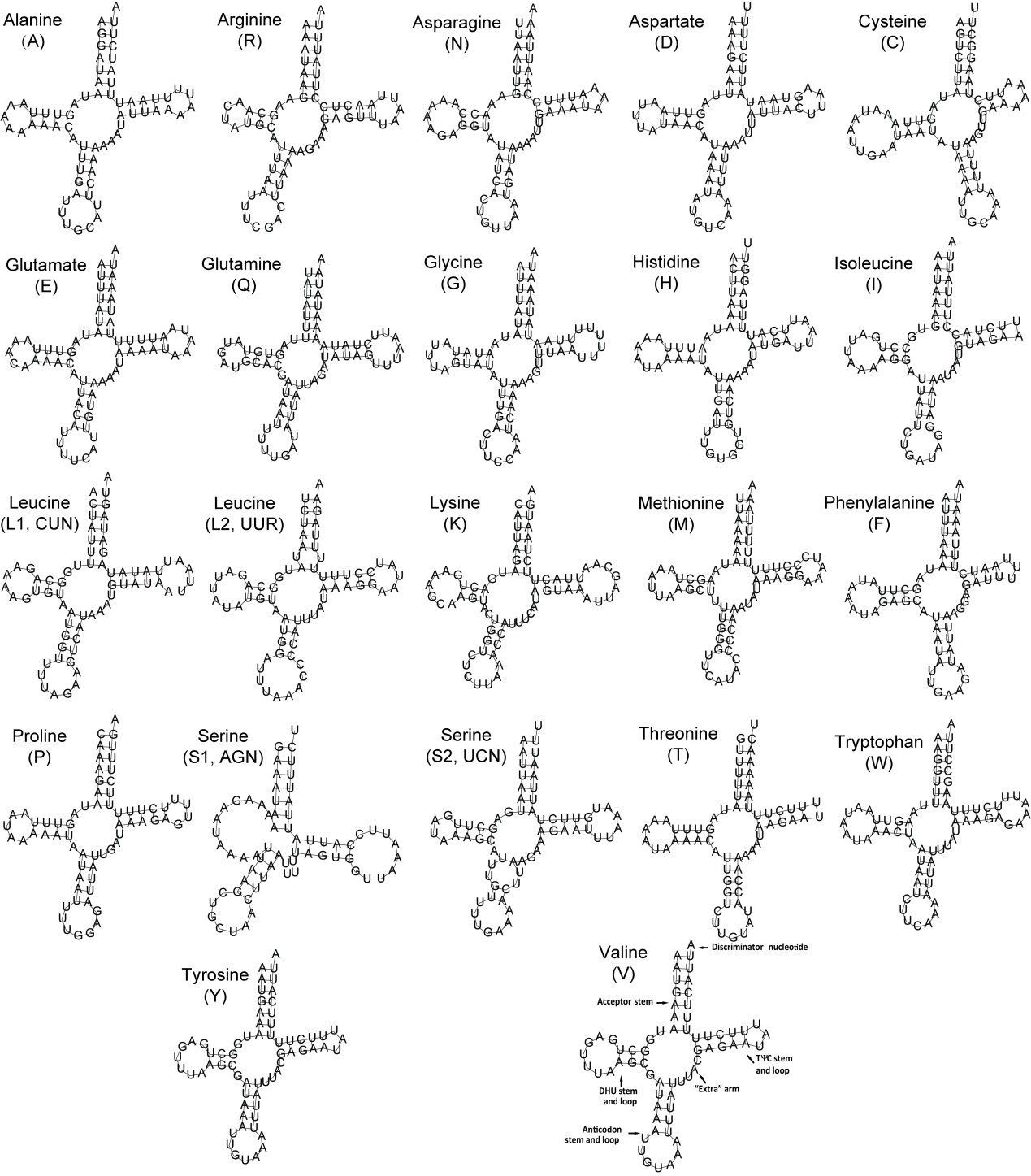


Figure S3. Secondary structures of 22 transfer RNAs in *Aethes alatavica*


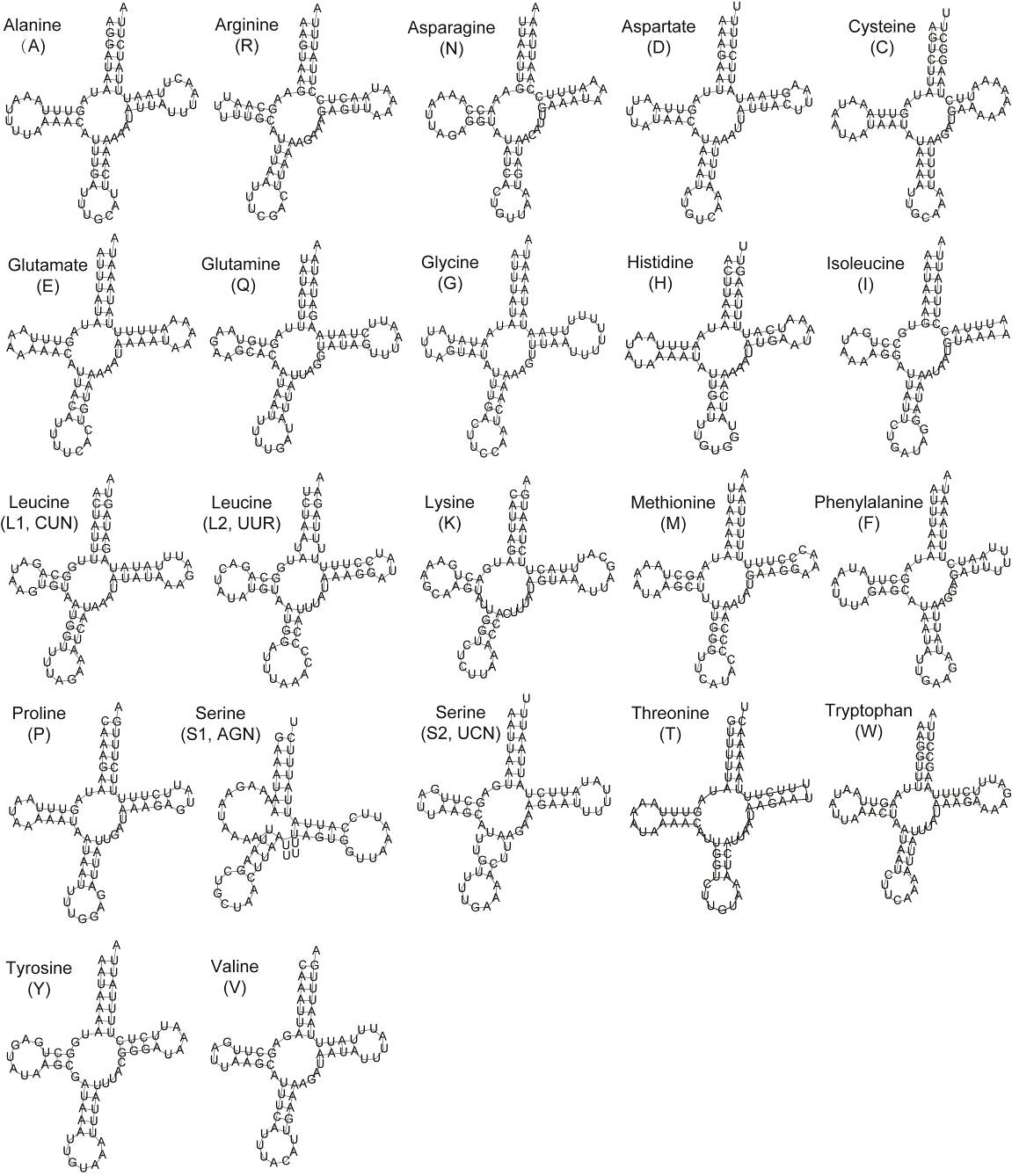


Figure S4. Secondary structures of 22 transfer RNAs in Cochylis faustana


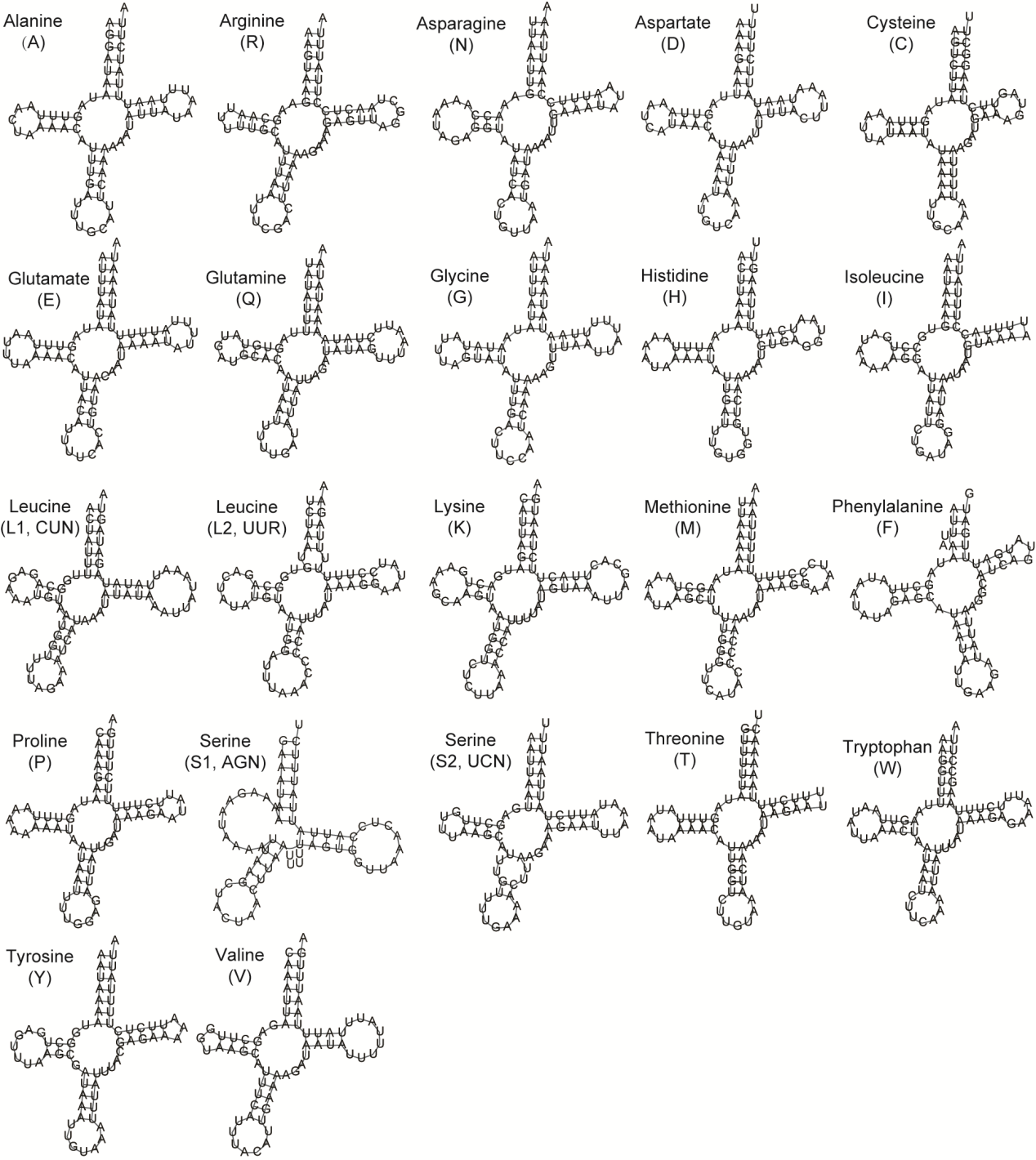


Figure S5. Secondary structures of 22 transfer RNAs in *Falseuncaria discerta* comb. nov.
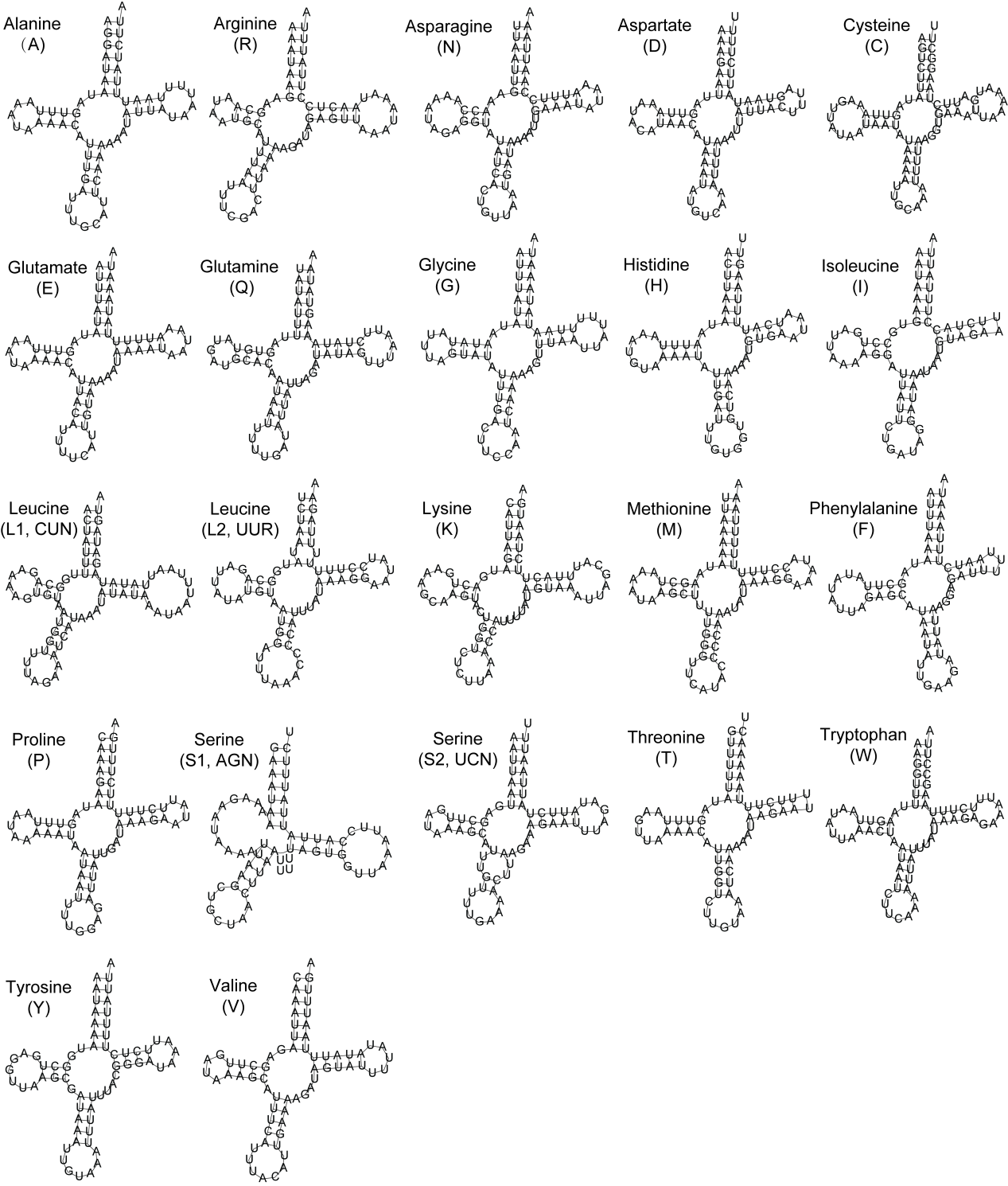


Figure S6. Secondary structures of 22 transfer RNAs in *Falseuncaria kaszabi*

Table S1. Collecting information of specimens in present study.

| **ID** | **Species** | **Genetalia Slide No.** | **Collecting sites** | **Collecting Date** | **Collectors** |
| --- | --- | --- | --- | --- | --- |
| S05 | *Aethes alatavica* (Danilevskij, 1962) | SYH007 | Yanchi County (37.76°N, 107.37°E), 1300m, Ningxia, China | 2019-VIII-21 | Yinghui Sun |
| S03 | *Falseuncaria discerta* comb. nov. | SYH005 | Yanchi County (37.76°N, 107.37°E), 1300m, Ningxia, China | 2019-VIII-21 | Yinghui Sun |
| S54 | Cochylis faustana (Kennel, 1919) | SYH048 | Mt. Xiaowutai, Yu County (39°30′N, 114°26′E)，1200 m，Hebei, China | 2018-VII-21 | Yinghui Sun |
| S06 | *Falseuncaria kaszabi* Razowski, 1966 | SYH008 | Yanchi County (37.76°N, 107.37°E), 1300m, Ningxia, China | 2019-VIII-21 | Yinghui Sun |

Table S2. Mitogenome organization of *Aethes alatavica*.

| **Name** | **Strand** | **Position (from)** | **Position (to)** | **Length** | **Anticodon** | **Start Codon** | **Stop Codon** | **Intergenic Nucleotides** |
| --- | --- | --- | --- | --- | --- | --- | --- | --- |
| trnM | J | 1 | 68 | 68 | CAT |  |  | 0 |
| trnI | J | 69 | 133 | 65 | GAT |  |  | -3 |
| trnQ | N | 199 | 131 | 69 | TTG |  |  | 55 |
| ND2 | J | 255 | 1265 | 1011 |  | ATT | TAA | -2 |
| trnW | J | 1264 | 1333 | 70 | TCA |  |  | -8 |
| trnC | N | 1392 | 1326 | 67 | GCA |  |  | 0 |
| trnY | N | 1457 | 1393 | 65 | GTA |  |  | 2 |
| COX1 | J | 1460 | 2996 | 1537 |  | TTG | T | -1 |
| trnL | J | 2996 | 3062 | 67 | TAA |  |  | 0 |
| COX2 | J | 3063 | 3744 | 682 |  | GTG | T | 0 |
| trnK | J | 3745 | 3815 | 71 | CTT |  |  | 10 |
| trnD | J | 3826 | 3891 | 66 | GTC |  |  | 0 |
| ATP8 | J | 3892 | 4048 | 157 |  | ATT | T | -2 |
| ATP6 | J | 4047 | 4724 | 678 |  | ATG | TAA | -1 |
| COX3 | J | 4724 | 5512 | 789 |  | ATG | TAA | 2 |
| trnG | J | 5515 | 5580 | 66 | TCC |  |  | 0 |
| ND3 | J | 5581 | 5934 | 354 |  | ATT | TAA | 39 |
| trnA | J | 5974 | 6038 | 65 | TGC |  |  | 0 |
| trnR | J | 6039 | 6103 | 65 | TCG |  |  | 1 |
| trnN | J | 6105 | 6170 | 66 | GTT |  |  | 3 |
| trnS | J | 6174 | 6239 | 66 | GCT |  |  | 0 |
| trnE | J | 6240 | 6306 | 67 | TTC |  |  | 86 |
| trnF | N | 6457 | 6393 | 65 | GAA |  |  | 0 |
| ND5 | N | 8189 | 6458 | 1732 |  | ATT | T | 0 |
| trnH | N | 8255 | 8190 | 66 | GTG |  |  | 0 |
| ND4 | N | 9594 | 8256 | 1339 |  | ATG | T | 0 |
| ND4L | N | 9885 | 9595 | 291 |  | ATG | TAG | 7 |
| trnT | J | 9893 | 9957 | 65 | TGT |  |  | 0 |
| trnP | N | 10023 | 9958 | 66 | TGG |  |  | 2 |
| ND6 | J | 10026 | 10553 | 528 |  | ATA | TAA | 10 |
| CYTB | J | 10564 | 11712 | 1149 |  | ATG | TAA | 2 |
| trnS | J | 11715 | 11781 | 67 | TGA |  |  | 15 |
| ND1 | N | 12732 | 11797 | 936 |  | ATG | TAA | 1 |
| trnL | N | 12800 | 12734 | 67 | TAG |  |  | 0 |
| l-rRNA | N | 14203 | 12801 | 1403 |  |  |  | 2 |
| trnV | N | 14271 | 14206 | 66 | TAC |  |  | 0 |
| s-rRNA | N | 15109 | 14272 | 838 |  |  |  | 0 |
| AT-rich region | J | 15110 | 15582 | 473 |  |  |  |  |

Table S3. Mitogenome organization of Cochylis faustana.

| **Name** | **Strand** | **Position (from)** | **Position (to)** | **Length** | **Anticodon** | **Start Codon** | **Stop Codon** | **Intergenic Nucleotides** |
| --- | --- | --- | --- | --- | --- | --- | --- | --- |
| trnM | J | 1 | 67 | 67 | CAT |  |  | 0 |
| trnI | J | 68 | 131 | 64 | GAT |  |  | -3 |
| trnQ | N | 197 | 129 | 69 | TTG |  |  | 61 |
| ND2 | J | 259 | 1269 | 1011 |  | ATC | TAA | -2 |
| trnW | J | 1268 | 1338 | 71 | TCA |  |  | -8 |
| trnC | N | 1399 | 1331 | 69 | GCA |  |  | 4 |
| trnY | N | 1468 | 1404 | 65 | GTA |  |  | 3 |
| COX1 | J | <1472 | 3003 | 1531 |  | CGA | T | -1 |
| trnL | J | 3003 | 3068 | 66 | TAA |  |  | 0 |
| COX2 | J | 3069 | 3750 | 682 |  | ATG | T | 0 |
| trnK | J | 3751 | 3821 | 71 | CTT |  |  | 3 |
| trnD | J | 3825 | 3890 | 66 | GTC |  |  | 0 |
| ATP8 | J | 3891 | 4047 | 157 |  | ATA | T | -2 |
| ATP6 | J | 4046 | 4723 | 678 |  | ATG | TAA | -1 |
| COX3 | J | 4723 | 5511 | 789 |  | ATG | TAA | 2 |
| trnG | J | 5514 | 5581 | 68 | TCC |  |  | 0 |
| ND3 | J | 5582 | 5935 | 354 |  | ATT | TAA | 43 |
| trnA | J | 5979 | 6038 | 60 | TGC |  |  | 9 |
| trnR | J | 6048 | 6113 | 66 | TCG |  |  | 2 |
| trnN | J | 6116 | 6183 | 68 | GTT |  |  | 57 |
| trnS | J | 6241 | 6306 | 66 | GCT |  |  | 0 |
| trnE | J | 6307 | 6372 | 66 | TTC |  |  | 28 |
| trnF | N | 6467 | 6401 | 67 | GAA |  |  | 0 |
| ND5 | N | 8170 | 6468 | 1703 |  | ATA | T | 0 |
| trnH | N | 8236 | 8171 | 66 | GTG |  |  | 0 |
| ND4 | N | 9575 | 8237 | 1339 |  | ATG | T | 0 |
| ND4L | N | 9866 | 9576 | 291 |  | ATG | TAA | 5 |
| trnT | J | 9872 | 9936 | 65 | TGT |  |  | 0 |
| trnP | N | 10002 | 9937 | 66 | TGG |  |  | 2 |
| ND6 | J | 10005 | 10535 | 531 |  | ATA | TAA | 10 |
| CYTB | J | 10546 | 11694 | 1149 |  | ATG | TAA | -2 |
| trnS | J | 11693 | 11759 | 67 | TGA |  |  | 16 |
| ND1 | N | 12711 | 11776 | 936 |  | ATG | TAA | 1 |
| trnL | N | 12779 | 12713 | 67 | TAG |  |  | 0 |
| l-rRNA | N | 14194 | 12780 | 1415 |  |  |  | 3 |
| trnV | N | 14262 | 14198 | 65 | TAC |  |  | 0 |
| s-rRNA | N | 15081 | 14263 | 819 |  |  |  | 0 |
| AT-rich region | J | 15082 | 15427 | 346 |  |  |  |  |

Table S4. Mitogenome organization of *Falseuncaria discerta* comb. nov.

| **Name** | **Strand** | **Position (from)** | **Position (to)** | **Length** | **Anticodon** | **Start Codon** | **Stop Codon** | **Intergenic Nucleotides** |
| --- | --- | --- | --- | --- | --- | --- | --- | --- |
| trnM | J | 1 | 68 | 68 | CAT |  |  | 0 |
| trnI | J | 69 | 133 | 65 | GAT |  |  | -3 |
| trnQ | N | 199 | 131 | 69 | TTG |  |  | 55 |
| ND2 | J | 260 | 1270 | 1011 |  | ATT | TAA | -2 |
| trnW | J | 1271 | 1340 | 70 | TCA |  |  | -8 |
| trnC | N | 1395 | 1333 | 63 | GCA |  |  | 0 |
| trnY | N | 1464 | 1400 | 65 | GTA |  |  | 2 |
| COX1 | J | 1468 | 2998 | 1531 |  | CGA | T | -1 |
| trnL | J | 2999 | 3065 | 67 | TAA |  |  | 0 |
| COX2 | J | 3066 | 3747 | 682 |  | ATG | T | 0 |
| trnK | J | 3748 | 3818 | 71 | CTT |  |  | 10 |
| trnD | J | 3825 | 3890 | 66 | GTC |  |  | 0 |
| ATP8 | J | 3891 | 4046 | 156 |  | ATT | TGA | -2 |
| ATP6 | J | 4043 | 4720 | 678 |  | ATG | TAA | -1 |
| COX3 | J | 4720 | 5508 | 789 |  | ATG | TAA | 2 |
| trnG | J | 5511 | 5576 | 66 | TCC |  |  | 0 |
| ND3 | J | 5577 | 5930 | 354 |  | ATT | TAA | 39 |
| trnA | J | 5957 | 6021 | 65 | TGC |  |  | 0 |
| trnR | J | 6026 | 6091 | 66 | TCG |  |  | 1 |
| trnN | J | 6115 | 6181 | 67 | GTT |  |  | 3 |
| trnS | J | 6191 | 6256 | 66 | GCT |  |  | 0 |
| trnE | J | 6257 | 6324 | 68 | TTC |  |  | 86 |
| trnF | N | 6415 | 6350 | 66 | GAA |  |  | 0 |
| ND5 | N | 8153 | 6420 | 1734 |  | ATT | T | 0 |
| trnH | N | 8219 | 8154 | 66 | GTG |  |  | 0 |
| ND4 | N | 9561 | 8220 | 1342 |  | ATG | T | 0 |
| ND4L | N | 9852 | 9562 | 291 |  | ATG | TAG | 7 |
| trnT | J | 9858 | 9922 | 65 | TGT |  |  | 0 |
| trnP | N | 9923 | 9988 | 66 | TGG |  |  | 2 |
| ND6 | J | 9991 | 10521 | 531 |  | ATG | TAA | 10 |
| CYTB | J | 10551 | 11699 | 1149 |  | ATG | TAA | 2 |
| trnS | J | 11698 | 11764 | 67 | TGA |  |  | 15 |
| ND1 | N | 12715 | 11780 | 936 |  | ATG | TAA | 1 |
| trnL | N | 12787 | 12717 | 71 | TAG |  |  | 0 |
| l-rRNA | N | 14175 | 12788 | 1388 |  |  |  | 2 |
| trnV | N | 14245 | 14180 | 66 | TAC |  |  | 0 |
| s-rRNA | N | 15056 | 14246 | 811 |  |  |  | 0 |
| AT-rich region | J | 15057 | 15330 | 274 |  |  |  |  |

Table S5. Mitogenome organization of *Falseuncaria kaszabi.*

| **Name** | **Strand** | **Position (from)** | **Position (to)** | **Length** | **Anticodon** | **Start Codon** | **Stop Codon** | **Intergenic Nucleotides** |
| --- | --- | --- | --- | --- | --- | --- | --- | --- |
| trnM | J | 1 | 68 | 68 | CAT |  |  | 0 |
| trnI | J | 69 | 133 | 65 | GAT |  |  | 65 |
| trnQ | N | 199 | 131 | 69 | TTG |  |  | 145 |
| ND2 | J | 277 | 1287 | 1011 |  | ATT | TAA | -2 |
| trnW | J | 1286 | 1355 | 70 | TCA |  |  | 64 |
| trnC | N | 1420 | 1348 | 73 | GCA |  |  | 140 |
| trnY | N | 1489 | 1425 | 65 | GTA |  |  | 67 |
| COX1 | J | 1493 | 3028 | 1536 |  | CGA | T | -5 |
| trnL | J | 3024 | 3090 | 67 | TAA |  |  | 0 |
| COX2 | J | 3091 | 3772 | 682 |  | ATG | T | 0 |
| trnK | J | 3773 | 3843 | 71 | CTT |  |  | 2 |
| trnD | J | 3846 | 3912 | 67 | GTC |  |  | 0 |
| ATP8 | J | 3913 | 4069 | 157 |  | ATT | T | -2 |
| ATP6 | J | 4068 | 4745 | 678 |  | ATG | TAA | -1 |
| COX3 | J | 4745 | 5533 | 789 |  | ATG | TAA | 2 |
| trnG | J | 5536 | 5601 | 66 | TCC |  |  | 0 |
| ND3 | J | 5602 | 5955 | 354 |  | ATT | TAA | 16 |
| trnA | J | 5972 | 6037 | 66 | TGC |  |  | -1 |
| trnR | J | 6037 | 6104 | 68 | TCG |  |  | 17 |
| trnN | J | 6122 | 6189 | 68 | GTT |  |  | 47 |
| trnS | J | 6237 | 6302 | 66 | GCT |  |  | 0 |
| trnE | J | 6303 | 6369 | 67 | TTC |  |  | 9 |
| trnF | N | 6444 | 6379 | 66 | GAA |  |  | -1 |
| ND5 | N | 8176 | 6444 | 1733 |  | ATT | TA | 0 |
| trnH | N | 8242 | 8177 | 66 | GTG |  |  | -1 |
| ND4 | N | 9581 | 8242 | 1340 |  | ATG | TA | 0 |
| ND4L | N | 9872 | 9582 | 291 |  | ATG | TAA | 5 |
| trnT | J | 9878 | 9942 | 65 | TGT |  |  | 0 |
| trnP | N | 10008 | 9943 | 66 | TGG |  |  | 2 |
| ND6 | J | 10011 | 10541 | 531 |  | ATG | TAA | 24 |
| CYTB | J | 10566 | 11714 | 1149 |  | ATG | TAA | -2 |
| trnS | J | 11713 | 11779 | 67 | TGA |  |  | 15 |
| ND1 | N | 12730 | 11795 | 936 |  | ATG | TAA | 1 |
| trnL | N | 12803 | 12732 | 72 | TAG |  |  | 8 |
| l-rRNA | N | 14218 | 12812 | 1407 |  |  |  | 4 |
| trnV | N | 14288 | 14223 | 66 | TAC |  |  | 0 |
| s-rRNA | N | 15108 | 14289 | 820 |  |  |  | 0 |
| AT-rich region | J | 15109 | 15426 | 318 |  |  |  |  |

Table S6. Nucleotide composition of mitochondrial genomes of four Cochylini species.

|  | *Aethes alatavica* | | | | | | | | |
| --- | --- | --- | --- | --- | --- | --- | --- | --- | --- |
| gene content | Length (bp) | T | C | A | G | AT | CG | ATskew | GCskew |
| Genome | 15482 | 0.399 | 0.114 | 0.405 | 0.082 | 0.804 | 0.196 | 0.007 | -0.166 |
| PCGs | 11181 | 0.450 | 0.102 | 0.336 | 0.112 | 0.786 | 0.214 | -0.145 | 0.048 |
| rRNAs | 2241 | 0.417 | 0.049 | 0.433 | 0.101 | 0.850 | 0.150 | 0.018 | 0.351 |
| tRNAs | 1468 | 0.394 | 0.080 | 0.416 | 0.110 | 0.810 | 0.190 | 0.026 | 0.161 |
| Control Region | 373 | 0.507 | 0.029 | 0.448 | 0.016 | 0.954 | 0.046 | -0.062 | -0.294 |
|  | *Cochylis faustana* | | | | | | | | |
| gene content | Length (bp) | T | C | A | G | AT | CG | ATskew | GCskew |
| Genome | 15427 | 0.401 | 0.113 | 0.407 | 0.079 | 0.808 | 0.192 | 0.008 | -0.175 |
| PCGs | 11152 | 0.452 | 0.101 | 0.339 | 0.108 | 0.791 | 0.209 | -0.144 | 0.037 |
| rRNAs | 2221 | 0.425 | 0.049 | 0.430 | 0.096 | 0.855 | 0.145 | 0.006 | 0.327 |
| tRNAs | 1465 | 0.394 | 0.078 | 0.419 | 0.109 | 0.813 | 0.187 | 0.031 | 0.161 |
| Control Region | 346 | 0.497 | 0.029 | 0.448 | 0.026 | 0.945 | 0.055 | -0.052 | -0.053 |
|  | *Falseuncaria discerta* comb. nov. | | | | | | | | |
| gene content | Length (bp) | T | C | A | G | AT | CG | ATskew | GCskew |
| Genome | 15330 | 0.399 | 0.115 | 0.405 | 0.081 | 0.804 | 0.196 | 0.008 | -0.171 |
| PCGs | 11187 | 0.451 | 0.101 | 0.339 | 0.110 | 0.789 | 0.211 | -0.142 | 0.039 |
| rRNAs | 2199 | 0.422 | 0.054 | 0.424 | 0.100 | 0.846 | 0.154 | 0.002 | 0.302 |
| tRNAs | 1469 | 0.395 | 0.081 | 0.415 | 0.110 | 0.809 | 0.191 | 0.024 | 0.150 |
| Control Region | 274 | 0.493 | 0.029 | 0.456 | 0.022 | 0.949 | 0.051 | -0.038 | -0.143 |
|  | *Falseuncaria kaszabi* | | | | | | | | |
| gene content | Length (bp) | T | C | A | G | AT | CG | ATskew | GCskew |
| Genome | 15462 | 0.396 | 0.117 | 0.406 | 0.080 | 0.802 | 0.198 | 0.013 | -0.187 |
| PCGs | 11187 | 0.448 | 0.104 | 0.336 | 0.112 | 0.784 | 0.216 | -0.143 | 0.036 |
| rRNAs | 2227 | 0.423 | 0.049 | 0.427 | 0.101 | 0.850 | 0.150 | 0.004 | 0.347 |
| tRNAs | 1484 | 0.393 | 0.076 | 0.420 | 0.111 | 0.813 | 0.187 | 0.033 | 0.187 |
| Control Region | 318 | 0.484 | 0.028 | 0.475 | 0.013 | 0.959 | 0.041 | -0.010 | -0.385 |

Table S7. The best substitute DNA model in Tortricidae using jModeltest.

| **Gene** | **Best substitute DNA model** |
| --- | --- |
| ATP6 | GTR+I+G |
| ATP8 | TIM3+I+G |
| COX1 | GTR+I+G |
| COX2 | GTR+I+G |
| COX3 | GTR+I+G |
| CYTB | GTR+I+G |
| ND1 | TVM+I+G |
| ND2 | TVM+I+G |
| ND3 | GTR+I+G |
| ND4 | GTR+I+G |
| ND4L | TVM+I+G |
| ND5 | TVM+I+G |
| ND6 | TVM+I+G |
