# Supplementary figures and images for: A New Taxonomic Placement of Cochylis discerta (Lepidoptera: Tortricidae) to Falseuncaria Supported by Congruent Mitogenomic and Morphological Evidence
Source: Ecol Evol. 2026 Jan 6;16(1):e72514. doi: 10.1002/ece3.72514 (PMC12771650; doi:10.1002/ece3.72514)

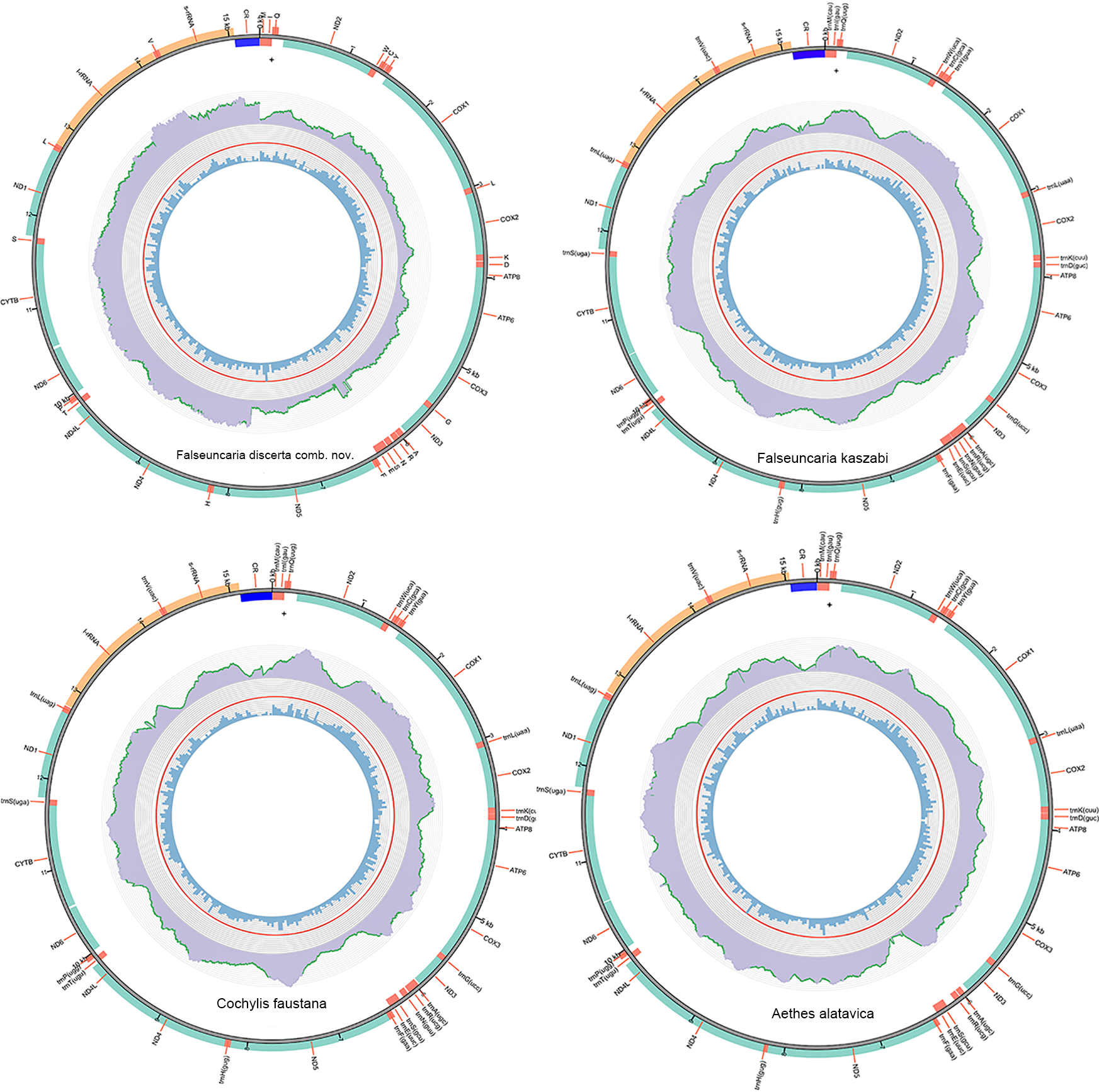

Supplement: Supplementary file 1 — Figure S1: Coverage plots of the four mitogenomes newly sequenced by this study. Figure S2: Sliding‐window nucleotide diversity of conserved amino‐acid sites across PCGs of Tortricidae. Figure S3: Secondary structures of 22 transfer RNAs in Aethes alatavica. Figure S4: Secondary structures of 22 transfer RNAs in Cochylis faustana. Figure S5: Secondary structures of 22 transfer RNAs in Falseuncaria discerta comb. nov. Figure S6: Secondary structures of 22 transfer RNAs in Falseuncaria kaszabi. Table S1: Collecting information of specimens in present study. Table S2: Mitogenome organization of Aethes alatavica. Table S3: Mitogenome organization of Cochylis faustana. Table S4: Mitogenome organization of Falseuncaria discerta comb. nov. Table S5: Mitogenome organization of Falseuncaria kaszabi. Table S6: Nucleotide composition of mitochondrial genomes of four Cochylini species. Table S7: The best substitute DNA model in Tortricidae using jModeltest. [file ECE3-16-e72514-s001.zip › ece372514-sup-0001-FigureS1@Figure S1. Coverage plots.tif]

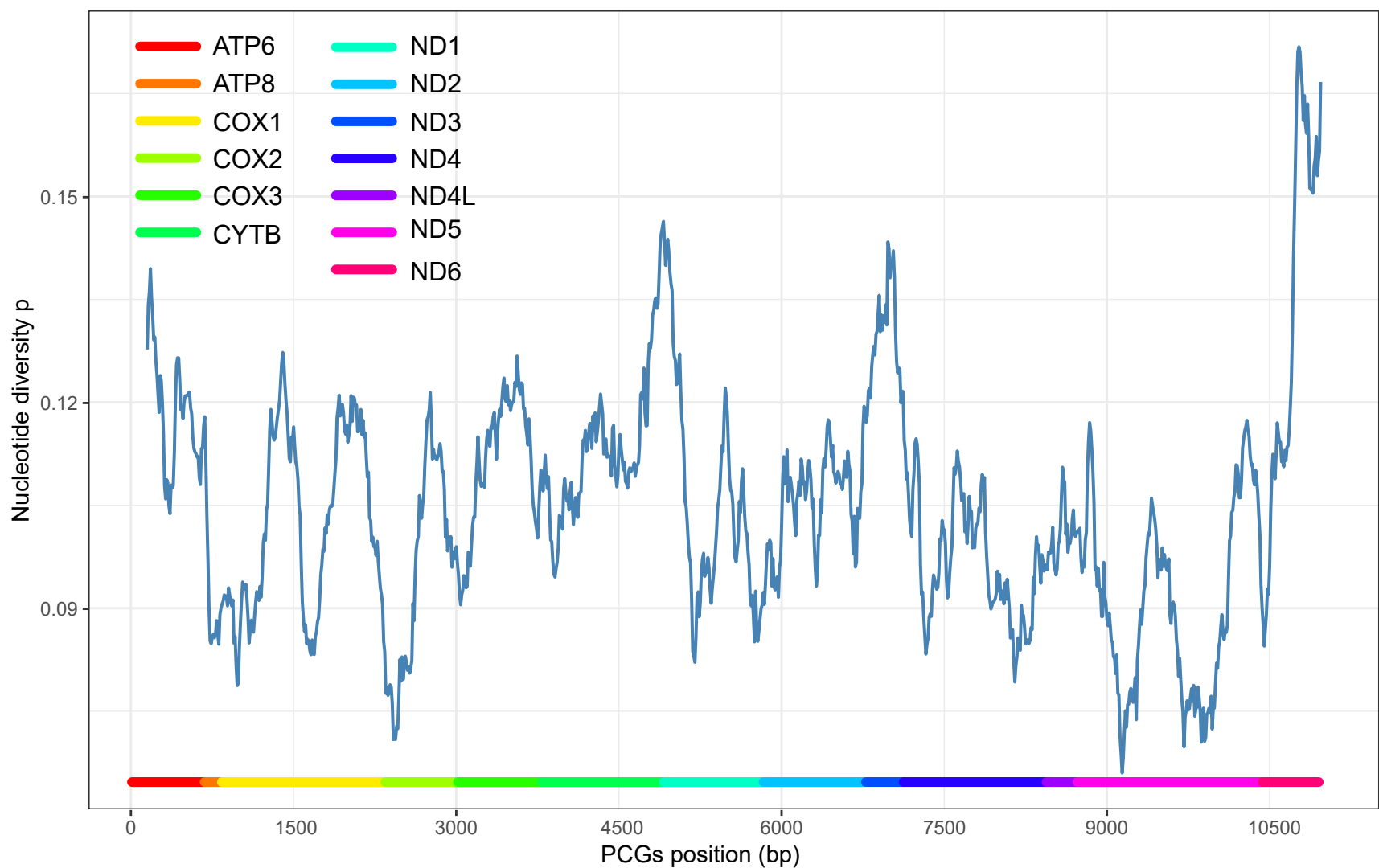

Supplement: Supplementary file 1 — Figure S1: Coverage plots of the four mitogenomes newly sequenced by this study. Figure S2: Sliding‐window nucleotide diversity of conserved amino‐acid sites across PCGs of Tortricidae. Figure S3: Secondary structures of 22 transfer RNAs in Aethes alatavica. Figure S4: Secondary structures of 22 transfer RNAs in Cochylis faustana. Figure S5: Secondary structures of 22 transfer RNAs in Falseuncaria discerta comb. nov. Figure S6: Secondary structures of 22 transfer RNAs in Falseuncaria kaszabi. Table S1: Collecting information of specimens in present study. Table S2: Mitogenome organization of Aethes alatavica. Table S3: Mitogenome organization of Cochylis faustana. Table S4: Mitogenome organization of Falseuncaria discerta comb. nov. Table S5: Mitogenome organization of Falseuncaria kaszabi. Table S6: Nucleotide composition of mitochondrial genomes of four Cochylini species. Table S7: The best substitute DNA model in Tortricidae using jModeltest. [file ECE3-16-e72514-s001.zip › ece372514-sup-0002-FigureS2@Figure S2. Sliding-window nucleotide diversity.pdf]
